# Faces and bodies in emotion recognition: complementary and conflicting cues

**DOI:** 10.3389/fpsyg.2026.1980859

**Published:** 2026-10-28

**Authors:** Yuanyi Peng, Alex Kafkas, Karen Lander

**Affiliations:** Division of Psychology, Communication and Human Neuroscience, University of Manchester, Manchester, United Kingdom

**Keywords:** body emotion, cue integration, cue reliability, emotion recognition, face emotion

## Abstract

The face has traditionally been considered the primary source of information for emotion recognition. However, in everyday social interaction, facial expressions are embedded within bodies, actions, scenes, and interactions. This review examines how facial and bodily expressions shape emotion recognition, focusing on when the two sources provide complementary or conflicting information. In most cases, face and body play complementary roles and can be integrated to facilitate emotion recognition, particularly when they convey congruent emotions or are presented dynamically. However, face-body integration may interfere when the two convey conflicting emotions. Here, recognition may be slowed, biassed, or shaped by the cue that provides more reliable evidence. When facial expressions are highly intense or ambiguous, the body may exert a stronger influence, whereas clear diagnostic facial cues, such as tears for sadness, may increase the influence of the face. We argue that face-body emotion perception is best understood through a flexible reliability-weighting account, in which observers weigh cues according to their clarity, diagnosticity, perceptual coherence, and relevance to the task. This weighting may vary across development, ageing, clinical conditions, individual differences, and cultural background, although current evidence often does not yet distinguish single-cue perception from altered cue integration.

## 1 Introduction

Research on emotion recognition has traditionally treated the face as the primary source of affective information (Ekman and O’Sullivan, 1988; Izard, 1994). Indeed, standard experimental tasks typically present stereotypical, posed facial expressions against blank backgrounds. However, outside the laboratory, facial expressions are embedded within body, visual, linguistic, social, and auditory contexts (Aviezer et al., 2017; Barrett et al., 2007; de Gelder et al., 2010; Hu et al., 2020; Wieser and Brosch, 2012; Straulino et al., 2023). This discrepancy between laboratory displays and everyday affective communication raises concerns about the ecological validity of face-only paradigms. As highlighted by Straulino et al. (2023), studying emotional expressions requires consideration of how expressive behaviour is produced and measured beyond conventional, posed facial displays. Importantly, although observable face and body movements may be associated with particular emotional states, they do not necessarily provide direct evidence of the emotion experienced by the individual. In this review, emotion recognition refers primarily to observers’ interpretation or categorisation of emotional information conveyed by facial and bodily cues, rather than direct measurement of the expresser’s subjective emotional experience. This mini review uses face-body emotion perception as a case for moving beyond isolated faces and towards more ecologically valid accounts of how people recognise emotion in whole persons and social scenes.

A key question concerns whether one source of information (face or body) exerts greater influence on emotion labelling than the other. The Theory of Constructed Emotion (Barrett, 2009, 2017) suggests that observers should rely most on whichever cue is most diagnostically useful, regardless of whether it is conveyed by the face or body. An alternative possibility, aligned with the basic emotion approach (e.g., Ekman, 1992), is that face expressions occupy a privileged position in emotion recognition and therefore receive greater emphasis than body information. Examining complementary and conflicting face-body cues helps to clarify when each source of information exerts greater influence on emotion recognition (Aviezer et al., 2008). Facial and bodily cues are considered emotionally congruent when they are perceived as conveying the same emotional category, such as a face expression associated with fear paired with a withdrawn body posture. Conversely, cues are considered incongruent when their perceived emotional meanings differ, such as when an angry face expression is paired with a fearful body posture. These classifications refer to the perceived emotional meanings assigned to expressions rather than necessarily reflecting the emotions experienced by the individuals displaying them. Early studies demonstrated that body context can alter the emotion attributed to a face, even when participants are instructed to ignore the body (see Aviezer et al., 2011; Meeren et al., 2005). Reviews and meta-analytic evidence indicate that faces and bodies mutually influence emotion perception and labelling (Lecker and Aviezer, 2021). However, the strength and direction of this influence depend on a number of factors such as cue clarity, emotion category, structural alignment, and task relevance (Abramson et al., 2017; Balas and Huynh, 2015; Lecker and Aviezer, 2021; Steward et al., 2025; Stienen and de Gelder, 2011).

Overall, observers appear to flexibly integrate facial and bodily information rather than assigning fixed priority to either source. The relative weight of each cue depends on how informative it is within the perceptual and task context. For example, a clear, dynamic face expression may outweigh body posture in one situation, whereas body emotion may exert greater influence on emotion judgements when the face expression is ambiguous. Understanding how observers resolve agreement and conflict between facial and bodily cues can clarify how emotions are recognised both in the laboratory and in the real world (Abramson et al., 2017). This context-sensitive integration helps explain why isolated, static, or decontextualised facial displays provide an incomplete account of emotion perception and why they should be complemented by designs that capture dynamic, embodied, and interactive affective communication.

## 2 Complementary face and body cues

When facial and bodily expressions agree, they provide congruent evidence about emotion (Aviezer et al., 2017; Poyo et al., 2018). Congruent combinations generally support faster and more accurate recognition than incongruent displays (Aviezer et al., 2008; Wang et al., 2017), and body information can help disambiguate weak or uncertain face expressions (Aviezer et al., 2012; Israelashvili et al., 2019). This benefit does not simply reflect the presence of an additional emotion cue; rather, face and body are combined during emotion perception, even at early stages of processing (Foster, 2022; Wang et al., 2017). There is an interactive but distributed system with connections amongst face-selective, body-selective, superior temporal, limbic, and action-related neural regions (Candidi et al., 2015; de Gelder et al., 2010; Foster, 2022; Hu et al., 2020).

Behavioural work suggests that this integration is difficult to suppress even when one cue is irrelevant to the task (Aviezer et al., 2011; Meeren et al., 2005; Oldrati et al., 2020). Although much of the literature focuses on body influences on face perception, faces also influence the emotion attributed to body expressions, indicating a bidirectional relationship between the two channels (Lecker et al., 2020). The shared signal hypothesis provides one account of this mutual influence (Albohn et al., 2022). Face and body expressions often carry common emotional information, including valence, arousal, and approach or withdrawal tendencies (Kret M. E. et al., 2013; Meeren et al., 2005; Willis et al., 2011). When the face and body cues agree, their shared information allows each to constrain the interpretation of the other. Rather than supporting a face-first model, these findings suggest a flexible weighting process in which the clearer or more diagnostic cue helps disambiguate the less informative one.

Complementary cues are particularly important in dynamic, naturalistic communication (Abramson et al., 2017; Chen and Whitney, 2021; de Gelder et al., 2010; Pollux et al., 2019). Unlike posed photographs, real-life expressions are less stereotypical, with movement and action revealing what a person is doing and what may happen next (Atkinson et al., 2004; Krumhuber et al., 2021, 2023; Van Der Zant and Nelson, 2021). Such dynamic information can facilitate the recognition of naturalistic postures (Van Der Zant and Nelson, 2021). For example, Abramson et al. (2017) found genuine face-body expressions of fear and anger were recognised more accurately than posed expressions. This influence of dynamic context is automatic and immediate (Chen and Whitney, 2021). Observers can rapidly track an unseen person’s changing emotion from surrounding events (Chen and Whitney, 2019, 2021). However, when the face is moving, attention can be redirected towards the face (Pollux et al., 2019). Beyond individual expressions, the orientation and movement of other people can also influence how an emotional expression is understood. Abramson et al. (2021) showed that whether individuals orient towards or away from one another influences recognition and that context determines emotion perception not only through emotional congruency but also through meaningful interactions between individuals. Complementary evidence can therefore arise within one person or across people, actions, and scenes.

An important methodological consideration is how face and body expressions are characterised and validated across studies. In the present mini review, we synthesise findings from studies employing different stimulus selection, coding and validation procedures. For example, Van Den Stock et al. (2007) conducted a pilot emotion-recognition study and selected bodily expressions with recognition accuracy above 70%. Similarly, Kret M. E. et al. (2013) selected facial and bodily expressions from established stimulus sets, retaining those with recognition accuracy above 80%. These approaches differ from the Facial Action Coding System (FACS), which describes observable facial movements (Ekman et al., 2002), and automated facial-expression analysis which can quantify aspects of expressive behaviour. In contrast, emotion-recognition tasks typically measure the emotional categories or meanings attributed to these expressions by observers. These approaches therefore assess related but distinct aspects of emotional communication. Distinguishing between the physical characteristics of expressions and their perceived emotional meaning is essential when interpreting evidence of face–body integration.

Complementary face-body cues can also guide broader social judgements. For example, face-body combinations influence perceptions of approachability, threat, and social status (Vincente et al., 2024; Willis et al., 2011). A relaxed body may make a mildly positive face seem more approachable, whereas a dominant or threatening posture may alter judgements even when the facial expression is unchanged (Willis et al., 2011). Congruent cues therefore show how affective signals can be pooled to support recognition and social evaluation. However, facilitation under agreement does not reveal how observers decide between cues when they convey different emotions. Incongruent displays provide a stronger test of cue weighting.

## 3 Conflicting face and body cues

The clearest evidence for the integration of facial and bodily emotion cues comes from incongruent displays, in which the two sources convey conflicting emotions. When the two cues differ, responses are usually slower, less accurate, or biassed towards the non-target cue (Chen et al., 2022; Kret M. et al., 2013). These resemble the Stroop interference effect (Botvinick et al., 2001) whereby information that should be ignored competes with the instructed judgement as its emotional meaning is processed automatically (Aviezer et al., 2011; Meeren et al., 2005). This interpretation is further supported by evidence of relatively early conflict detection, reflected in the N2 ERP component (Chen et al., 2022), and the persistence of interference even when emotion is not the explicit focus of attention (Oldrati et al., 2020). Conflicting cues are therefore not resolved only through deliberate reasoning after perception, rather conflict emerges during the formation of the percept itself.

The relative influence of facial and bodily cues cannot be explained by a simple face-first rule (Aviezer et al., 2011; Van Den Stock et al., 2007). Instead, it depends on the clarity and diagnostic value of the emotional information conveyed by each source. When facial expressions are weak, ambiguous, or misleading, bodily information may become more influential in emotion recognition (Van Den Stock et al., 2007; Karaaslan et al., 2020; Albohn et al., 2022). Consistent with this account, Steward et al. (2025) identified signal clarity as a major moderator of face-body integration. When the face does not strongly distinguish amongst possible emotions, posture and action provide additional evidence for resolving uncertainty. Reduced facial diagnosticity is not limited to low-intensity expressions. At extreme intensities, positive and negative facial expressions can become visually similar (Aviezer et al., 2012). Xiao et al. (2016) found that observers could not reliably distinguish winning from losing at peak-intensity facial expressions. In these cases, the body may provide additional action and situational information that helps resolve this ambiguity (Israelashvili et al., 2019). However, observers may still attribute their emotional judgements to the face, even when these are primarily driven by bodily information, a phenomenon known as illusory facial affect (Aviezer et al., 2012). Neural evidence further suggests that face-body interaction under high-valence conditions involved multiple stages of processing, with modulation of the P1, N170, N2, and P3 ERP components (Wang et al., 2017). Thus, increasing facial-expression intensity may not necessarily improve recognition. At extreme intensities, bodily information may become a more reliable cue.

Taken together, when facial cues are ambiguous, observers rely more on body information, whereas clearer and more diagnostic facial cues may reduce this reliance. For example, visible tears reduced the influence of body posture, scenes, and written scenarios by enhancing the diagnostic value of facial information (Ito and Ong, 2023). Thus, signal clarity, as discussed by Steward et al. (2025), may reflect not only the visual distinctiveness of expression cues but also how clearly they communicate emotional meaning to the observer. Beyond cue clarity, the emotional category may also influence perception, as different emotions receive different levels of perceptual priority. For example, fear, which signals danger and withdrawal, may influence face-body integration differently from other emotions (Bertini and Làdavas, 2021). Chen et al. (2022) found that fearful body expressions produced greater interference on faces than happy body expressions. Similarly, Stienen and de Gelder (2011) found that fear-related information tended to dominate awareness regardless of whether it was conveyed by the face or body, with the right posterior superior temporal sulcus implicated in regulating their relative dominance (Candidi et al., 2015). Anger may show a similar effect to fear. Comparing six basic emotions, Martinez et al. (2016) found that anger was the only emotion for which bodily expressions yielded recognition accuracy comparable to facial expressions, whereas faces generally showed an advantage over bodies for other emotions. Both fear and anger are characterised by negative valence and high arousal (Russell, 1980), and are associated with threat-related responses, like fight-or-flight reactions (Springer et al., 2007). These shared characteristics may increase the perceptual salience of their emotional meaning, rather than necessarily enhancing the visual clarity of their expression cues. Therefore, the relative dominance of face and body may depend not only on the diagnosticity of their expressions but also on the emotional significance of the information they convey.

Beyond cue clarity, face-body integration also depends on whether the two sources are perceived as belonging to the same person and event. When facial and bodily expressions are naturally aligned, they are more readily integrated, strengthening shared emotional meaning and semantic-label effects (Lecker and Aviezer, 2021). However, this integration can be weakened by disruptions to spatial configuration or temporal synchrony. For example, inversion or disrupted configurations may reduce processing efficiency, as face emotion relies more on horizontal information and body emotion more on vertical information (Balas and Huynh, 2015), whilst temporal delays between facial and bodily expressions can decrease their integration (Lecker et al., 2017). Beyond perceptual organisation, task demands may further determine which source receives greater weight. For example, face may be useful for identifying individuals, whilst the body may be more diagnostic of threat, status, or approach readiness (Vincente et al., 2024). This influence extends beyond emotion recognition, as bodily expressions can affect facial identity matching and memory for faces (Van Den Stock and de Gelder, 2012, 2014). Together, these findings are consistent with a flexible reliability-weighting account (Goel et al., 2024), in which the relative influence of face and body depends on several interacting sources of evidence including how clearly each cue is visible, how strongly it distinguishes the relevant emotion, whether the cues are structurally and temporally aligned, whether they can be assigned to the same person and event, and whether they are relevant to the observer’s current goal. When faces and bodies agree, these forms of evidence can be pooled. When they disagree, the channel with the stronger combination of clarity, diagnosticity and relevance exerts greater influence. This account explains why body context can dominate one display whilst facial emotion dominates another, without requiring a universal hierarchy between the two.

## 4 Observer factors and broader implications

The integration of facial and bodily emotion cues appears to emerge early in development, although its relative weighting may change with age (Lecker and Aviezer, 2025). By 8 months of age, bodily expressions can influence facial emotion processing (Rajhans et al., 2016), and infant neural responses show integration of face emotion with action kinematics (Addabbo et al., 2026). This integration continues into childhood and adolescence, with studies demonstrating that children under the age of 18 years combine emotional information from faces and bodies (Nelson and Mondloch, 2017; Nelson and Russell, 2012; Nguyen and Nelson, 2021). In individuals aged 8–18 years, microstructural properties of specific white-matter pathways are associated with facial-expression and body-posture perception, as well as with the influence of bodily context on facial-expression judgements (Ward et al., 2023). These findings suggest that face-body emotion integration is present from infancy onwards, although the available evidence does not yet establish a detailed developmental trajectory for cue weighting. In particular, it remains unclear whether age-related changes reflect improvements in recognising individual facial or bodily cues, changes in the relative reliability assigned to each channel, or increasing ability to resolve conflict between cues. It is possible, for example, that the relative primacy of facial information changes as children’s understanding of facial expressions becomes increasingly differentiated and their capacity to integrate multiple emotion signals matures (Leitzke and Pollak, 2016; Theurel et al., 2016). Longitudinal or developmentally stratified studies that manipulate cue clarity, congruence, and task demands would help determine whether children’s apparent use of body context reflects mature cue integration or transitional strategies for managing uncertain facial information.

At the other end of the lifespan, ageing research demonstrates the limitations of face-only tasks. Older adults often rely more heavily on bodily context than younger adults when face and body expressions disagree (Abo Foul et al., 2018; Noh and Isaacowitz, 2013). This may compensate for reduced sensitivity to face detail, but it may also reflect greater use of holistic or socially meaningful information (Isaacowitz and Stanley, 2011; Meinhardt-Injac et al., 2014). When displays are dynamic and the whole person is visible, age differences can be substantially reduced (Abo Foul et al., 2022). These findings suggest that older adults’ performance depends strongly on the format and ecological validity of the task, including whether facial cues are presented in isolation or alongside posture, movement, and action information. Apparent problems in laboratory facial recognition may therefore overstate difficulties in everyday communication (Isaacowitz and Stanley, 2011), where additional posture and movement information are available. Further work should clarify whether age-related differences reflect changes in single-cue sensitivity, altered reliability weighting across cues, or strategic use of richer person-level information.

Clinical studies also reveal distinct alterations in the use of emotion cues. In Huntington’s disease, explicit recognition of facial expressions can be impaired whilst sensitivity to body and situational context remains relatively preserved. This suggests context may still shape emotion perception despite difficulties identifying facial expressions directly (Aviezer et al., 2009). Further, people with Parkinson’s disease may rely more on the body when facial and bodily expressions conflict, whilst priming findings indicate difficulties in the automatic integration of information across the two cues (Abo Foul et al., 2024; Longo et al., 2025). Frontotemporal dementia also alters the extent and manner in which contextual information modulates facial-emotion judgements, with potentially distinct patterns across clinical subtypes (Kumfor et al., 2018). These examples show that clinical populations may differ in cue use, but they do not support a uniform interpretation in terms of impaired integration. As many of these studies assessed different channels separately, further research is required to establish whether the observed difficulties arise from impaired perception within a particular channel, atypical weighting of multiple signals, or difficulty benefiting from agreement and resolving conflict between them. Clinical assessment should therefore distinguish poor perception of an individual cue from altered integration across cues, and should consider whether apparent difficulties depend on task format, cue clarity, and availability of contextual information.

Finally, cultural evidence remains less extensive, but cautions against assuming that all aspects of cue weighting are universal. Witkower et al. (2021) found substantial recognition of bodily expressions in the relatively isolated Mayangna community, supporting cross-cultural sensitivity to some body signals. However, evidence for shared recognition of some bodily expressions does not imply that all postures, actions, or face-body combinations carry the same meaning across cultural settings. Broad affective dimensions such as valence and arousal may be widely shared, whilst the social meaning attached to a particular posture or face-body combination may depend on cultural experience. Future cultural research should therefore use diverse participant groups and culturally grounded stimuli and should distinguish cross-cultural sensitivity to broad affective signals from culturally learned interpretations of whole-person behaviour.

## 5 Discussion

Overall, faces and bodies make distinct but interdependent contributions to emotion recognition. Their agreement provides complementary evidence that can clarify ambiguous expressions, support naturalistic understanding, and guide judgements about intention and action. Their disagreement creates perceptual competition, revealing that neither channel is uniformly dominant. The central conclusion of this review is therefore that face-body emotion perception follows a flexible reliability-weighting principle. Observers flexibly weigh the cue that is clearest, most diagnostic, structurally coherent, and relevant to the immediate goal. Development, age, culture, and clinical condition can alter this balance by changing cue availability, cue interpretability, or the strategies observers use to resolve uncertainty. Beyond diagnostic group differences, future studies could test whether individual characteristics, including social anxiety and empathy, predict reliance on bodily information (Lynn et al., 2018; Olderbak and Wilhelm, 2017). This could help establish whether variation in emotion-cue integration contributes to differences in emotion perception within, as well as between, clinical groups (Kumfor et al., 2018).

Finally, future research also needs to connect emotion recognition with the objective measurement of expressive behaviour and the timing and neural organisation of cue integration. Three-dimensional (3-D) kinematic analysis offers a valuable approach for quantifying the spatial and temporal characteristics of expressive movements. Recent studies have demonstrated its potential for investigating spontaneous facial expressions of happiness (Straulino et al., 2025) and differences between posed and spontaneous facial expressions of fear and surprise (Scarpazza et al., 2026). These findings further highlight the importance of distinguishing the measurable physical characteristics of expressive movements from the emotional meaning attributed to them by observers. Such approaches complement EEG/ERP, eye tracking, physiological measures and neuroimaging, which provide insights into different aspects of emotion processing and cue integration (Addabbo et al., 2026; Aviezer et al., 2008; Candidi et al., 2015; Chen et al., 2022; Hu et al., 2020; Kret M. et al., 2013). Combining these measures with observers’ judgements could help establish which face and body features predict perceived congruence and the relative influence of each cue.

Future accounts should predict when agreement facilitates perception, when conflict is detected, how attention is distributed, and how a final person-level judgement emerges. They should also extend beyond full faces and bodies: hands integrate automatically with facial emotion (Longo and Mackenzie, 2026), whilst bodily cues interact with voices, language, scenes, and other people. Future models therefore need to consider how information from multiple channels is integrated across different stages of emotion perception. In conclusion, framing the literature in terms of complementary and conflicting cues provides an accurate account of affective communication: emotion is recognised through the relationship between signals, not from the face or body alone.
